# The Role of ES&T in Advancing Environmental Toxicology and Chemical Risk Assessment: Past, Present, and Future

**DOI:** 10.1021/acs.est.6c03315

**Published:** 2026-06-01

**Authors:** Beate I. Escher, Joop L. M. Hermens, John P. Sumpter, Gerald T. Ankley

**Affiliations:** † Department of Cell Toxicology, Helmholtz Centre for Environmental Research − UFZ, Permoser Str. 15, Leipzig 04318, Germany; ‡ Environmental Toxicology, Department of Geosciences, Eberhard Karls University Tübingen, Schnarrenberger Str. 94-96, Tübingen 72076, Germany; § German Center for Child and Adolescent Health (DZKJ), partner site Leipzig/Dresden, Leipzig 04103, Germany; ∥ Institute for Risk Assessment Sciences, 8125 Utrecht University, Utrecht 3508 TD, The Netherlands; ⊥ Brunel University London, Uxbridge, Middlesex UB8 3PH, U.K.; # Integrated Biological Sciences, Department of Biology, University of Minnesota, Duluth, Minnesota 55812, United States

**Keywords:** ecotoxicology, new approach methodologies (NAM), (eco)exposome, adverse outcome pathway (AOP), aggregate exposure pathway (AEP), chemical hazard, exposure, toxicokinetic−toxicodynamic models, one health

## Abstract

Environmental contamination poses risks to all components of the ecosystem—humans and wildlife—yet toxicological research and regulatory assessment remain largely compartmentalized by discipline and organismal focus. We look back on 60 years of toxicological research published in *Environmental Science and Technology* (*ES&T*) and analyze how the field has evolved, what role *ES&T* has played in this evolution, and suggest a path forward for the future. Chemicals, complex mixtures, and their transformation products act across interconnected biological taxa, including humans, that share conserved molecular and physiological pathways. Integrating ecotoxicology, human toxicology, exposomics, and data-driven new approach methodologies can shift hazard and risk assessment from single-chemical, single-species paradigms toward a mechanism-based, systemic understanding of toxicity across the entire ecosystem. We discuss advances in the characterization of adverse outcome pathways and key biological targets, mixture-oriented testing strategies with effect-based bioassays, and advanced computational approaches. Understanding shared and specific toxicity pathways enables earlier and more reliable detection of potential chemical hazards, strengthens cross-species extrapolation, and supports the development of more predictive and sustainable chemical design and management strategies in the context of the One Health paradigm.

## Graphical abstract

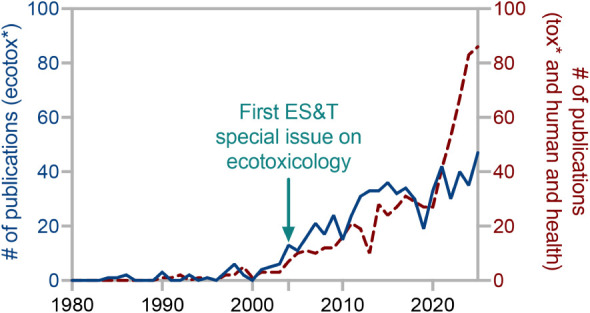

## Introduction

*Environmental Science and Technology* (*ES&T*) has been a thought leader in environmental sciences for 60 years. *ES&T* has evolved into a multidisciplinary environmental science journal, covering environmental chemistry, fate and transport, exposure science, human and ecosystem health connections, and ecological effects of contaminants, with a scope that explicitly includes research on the effects of chemicals on organisms and ecosystems. Synthetic chemicals and pollution were the prime *ES&T* publication topics in the early years with the impacts of contaminants (i.e., toxicity) forming only a very small fraction of published papers until the mid-1990s, when publications in these areas started to rapidly increase (Supporting Information, Text S1). Initially, hazard and risk considerations were only a motivation for published research but increasingly they became the center of the actual research, including policy analysis^1^ and socio-economic aspects of chemical pollution.^2^

Over the past two decades the four of us have served as Associate or Special Editors for *ES&T* in the field of ecotoxicology. In 2004, a special issue entirely devoted to ecotoxicology (Volume 38 (23)) was published with an editorial^3^ by the guest editors clearly demonstrating the timeliness, scope and impact of ecotoxicological research in *ES&T*. There was a subsequent increase in *ES&T* publications on ecotoxicological topics, with the numbers plateauing during the 2010s but with a steady increase in the number of *ES&T* publications focused on human health and toxicology (Text S1). The editorial^4^ for the special issue on “The Exposome and Human Health” in 2025 is a testimony of a move toward inclusion of human oriented-studies on pollution. Irrespective of the topic human health or ecotoxicology—the impact of *ES&T* publications has steadily increased in terms of citation numbers.

In the 2004 special issue editorial^3^ we noted “*Mechanism- and process-oriented studies are critical to ecotoxicology, providing detailed insights (e.g., at the molecular level) that form the basis for extrapolation across chemicals, species, and, ultimately, ecosystems.*” Substantial progress has been made in the field on this topic, with *ES&T* making significant contributions. For example, a perspectives article in the journal was one of the first to systematically describe the application of omics to risk assessment and regulation in ecotoxicology,^5^ and a focus issue on environmental genomics was published in 2012.^6^ Conventional ecological studies have not been a past area of emphasis in *ES&T*, although there have been publications highlighting critical concepts in the context of stress ecology.^7^ Scores of additional papers published in *ES&T* over the last 20 years have described and highlighted the use of a variety of new approach methodologies (NAMs), including in vivo omics, high throughput (HT) in vitro assays and integrated computational models, to support ecological risk assessment. NAMs are proving critical to helping assess the tens of thousands of chemicals (Figure 1) that have been highlighted as potential concerns since the early 2000s.^8,9^ Cost-effective NAM-based screening tools complement detailed chemical investigations in environmental quality assessment and chemical hazard assessment. This is especially relevant for industrial chemicals that are not intentionally potent^10^ and therefore less likely to cause effects via highly specific mechanisms of action (MOA) as opposed to baseline toxicity (also termed narcosis)^11–13^ (Figure 1). This contrasts with pesticides, biocides and pharmaceuticals that are designed for their bioactivity and therefore may cause strong target and unwanted nontarget effects and require research at higher levels of biological organization including, where appropriate, ecosystem level and behavioral studies^14^ (Figure 1). Given the scope of the challenge faced in terms of number of chemicals (and mixtures) of possible concern, hazard assessments focused on ecotoxicology and human toxicology should be focused, when possible, on conserved toxicity pathways even if they lead to different taxa-specific outcomes.

**Figure 1:**
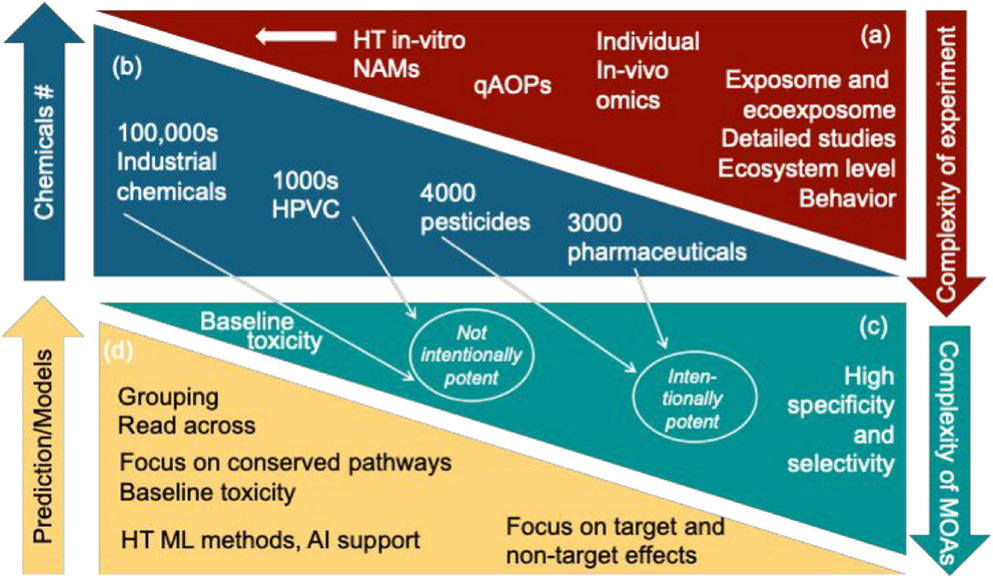
(a) Experimental approaches used for assessing effects in toxicology range from high throughput (HT) in vitro assays and other new approach methodologies (NAMs) to ecosystem-level analyses; (b) The large number of chemicals can be divided into industrial chemicals of which approximately 1000 are high production volume chemicals (HPVC) and a lesser number of pesticides and pharmaceuticals (c) Industrial chemicals are not intentionally potent^10^ and often act at baseline-toxic concentrations on diverse end points, while pesticides and pharmaceuticals are intentionally potent^10^ and typically have a high specificity and selectivity of their MOA. (d) Prediction methods and models have different foci depending on the type of chemicals. qAOP = quantitative adverse outcome pathways, NAM = new approach methodologies, MOA = Mode of action, HT = high throughput, ML = machine learning, AI = artificial intelligence.

## Mechanistic View on (Eco)toxicology

NAMs are the future of toxicology. Most NAMs involve the measurement or prediction of molecular, biochemical, or physiological effects that reflect chemical mechanisms. In addition to generating data efficiently, these tools can address major extrapolation challenges, such as those across chemical structures, among different taxa of concern, and across biological levels of organization. This includes populations and communities that are critical to the structure and function of sustainable ecosystems. In nearly every issue of *ES&T* there are multiple papers focused on the development or application of NAMs that viably could enhance toxicological risk assessments in humans and wildlife. Yet the adoption and use of NAM tools and data by risk assessors and regulators have been very limited. Part of this involves innate organizational resistance to change,^15^ but a critical conceptual technical concern with the use of NAMs involves uncertainties concerning the linkage of biological changes suggested by mechanistic data and apical adverse outcomes (AO). To address the lack of transparent, causal linkages between NAM data and end points related to survival, growth and reproduction, the adverse outcome pathway (AOP) framework was proposed as a communication and data translation tool to facilitate regulatory decision-making.^16^ An AOP provides a systematic, weight-of-evidence based approach linking molecular initiating events (MIE) to intermediate key events (KE) that span biological levels of organization culminating in an AO. The AOP concept has been advanced and utilized internationally through the efforts of groups such as the Organization for Economic Cooperation and Development (OECD) as key to facilitating and harmonizing use of NAM data for chemical safety assessments.^17^

*ES&T* has been a strong supporter of the AOP framework,^16^ with 160 articles touching on this concept.^18^ Many of these papers have involved expansion of the AOP framework in terms of flexibility and application. For example, from simple linear qualitative depictions of biological processes, AOPs can be developed as more complex networks suitable for addressing specific regulatory demands,^19,20^ and also can provide model-based quantitative predictions of relevant AOs (qAOPs) based on NAM data that measure MIEs or early in vivo KEs.^21^ Consequently, qAOPs are important to prediction of the probability of apical effects based on data from new NAMs such as HT in vitro assays or in vivo genomic changes.

### Importance of Understanding Exposure

The AOP framework deals with the biological side of adverse effects. It does not include the exposure and uptake into organisms in the environment and the toxicokinetic behavior inside organisms. The hazard and exposure characteristics together will determine the overall adverse toxicological effects of a contaminant. Analogous to the AOP framework, the Aggregate Exposure Pathway (AEP) framework was proposed by Teeguarden et al.^22^ The AEP combines multiple sources and exposure pathways in the estimation of the internal target exposure. The target exposure is estimated from the external dose and characteristics related to absorption, distribution, metabolism and elimination (ADME). The internal exposure forms the crucial link between the AEP and AOP framework^22^ (Figure 2). In the same line of reasoning, Conolly et al.^21^ stated that a qAOP is not chemical-specific but that by including ADME properties together with the relative potency of the interaction with the target (the MIE level), a qAOP can be used for many different chemicals (Figure 2). Toxicokinetic–toxicodynamic (TK/TD) models complement qAOPs by explicitly capturing ADME.^24^

**Figure 2:**
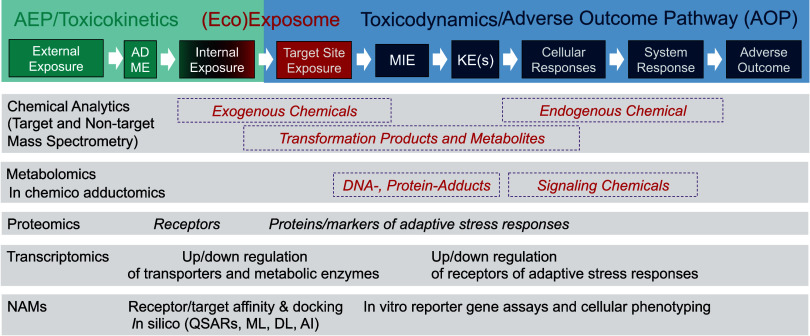
Interface between toxicokinetics (TK, teal) and toxicodynamics (TD, blue) connecting the aggregate exposure pathway (AEP) with the adverse outcome pathway (AOP) and how these concepts intercept with the (eco)­exposome (in red). The red dashed boxes represent chemical components of the exposome. ADME stands for the TK processes of absorption, distribution, metabolism and elimination. The gray boxes indicate experimental methods and models to quantify the chemical components of the exposome and the biological components of the AOP using new approach methodologies (NAMs). Figure adapted from Teeguarden et al.^22^ and Escher et al.^23^.

Essential in connecting external contaminant exposure to AOPs is the internal exposure concentration, or dose (Figure 3a).^22,23^ The exposome describes the internal exposure to pollutants but includes also endogenous chemicals and metabolites that are produced or altered in response to external stressors.^25,26^ Initially framed for human exposure,^27^ the concept has been generalized and extended to wildlife, the so-called ecoexposome.^28^ The exposome per definition accounts for complex mixtures and therefore nontargeted exposomics^29^ has been complemented by effect-based tools to quantify mixture effects of chemical cocktails extracted from environmental organisms, e.g., marine mammals^30^ or fish^31^ and in effects-based human biomonitoring.^32,33^ By using mixture indicators to quantify the exposome, we are gaining a truer understanding of the complexity of real-life mixtures; further, the application of NAMs in exposomics is a path forward to better determine effect-scaled concentrations.^13^

**Figure 3:**
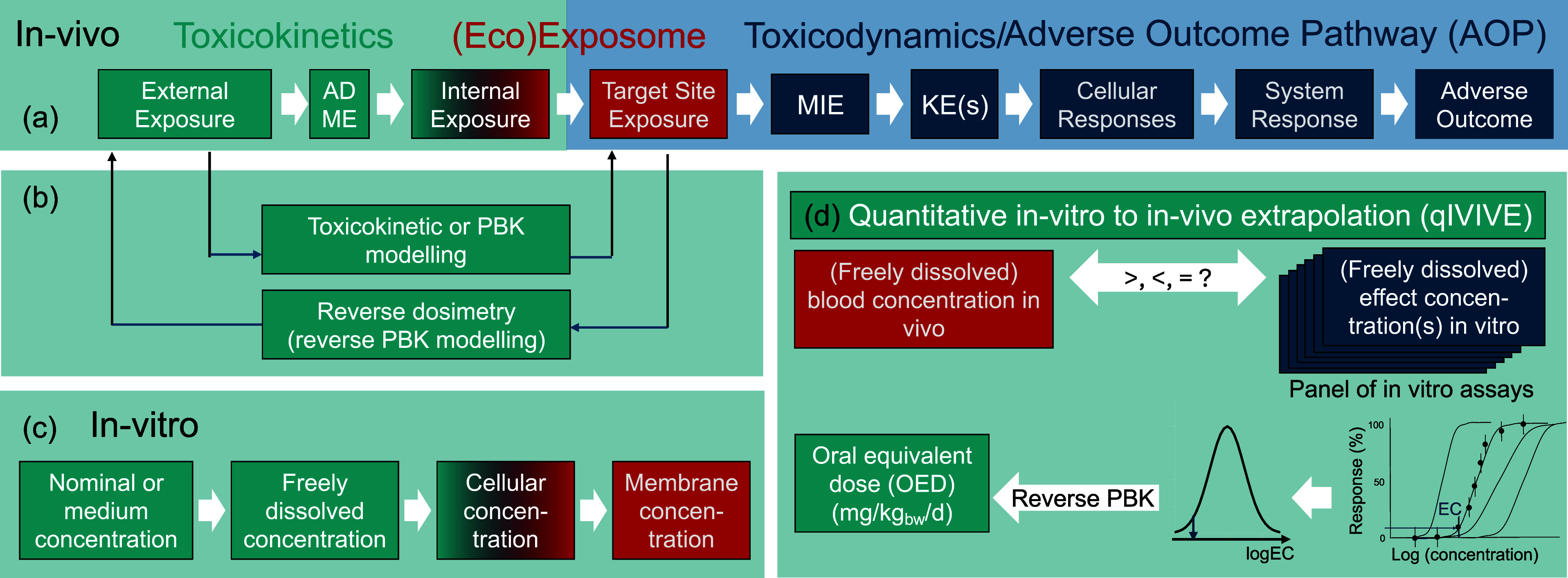
(a) Role of toxicokinetics (TK, teal) and toxicodynamics (TD, blue) for understanding the (eco)­exposome and adverse outcome pathways (AOP). (b) TK or physiologically based kinetic (PBK) modeling to predict internal and target site exposure in vivo.^43^ (c) In vitro exposure metrics ranging from nominal concentrations over freely dissolved concentrations to cellular and target site (e.g., membrane or cytosol) concentrations.^44^ (d) qIVIVE models either compare the (freely dissolved) blood concentrations with the (freely dissolved) effect concentrations in in vitro assays^45,46^ or use a lower percentile of the distribution of effect concentrations to derive an oral equivalent dose (OED), by reverse PBK modeling.^34^

The internal dose, represented as the internal concentration or target concentration, can be estimated from the (nominal or measured) external exposure, a physiologically based kinetic (PBK) model, and ADME properties (Figure 3b). The cellular in vivo response is directly related to this internal exposure and the potency of interaction of the specific chemical with the target. Further, PBK modeling can also be applied to the prediction of external exposure based on internal exposure (reverse dosimetry).^34^ In a qAOP that is established for a certain MIE and a reference chemical, reverse dosimetry is useful to estimate a toxicological reference dose (or concentration) for related chemicals. The only input needed is information for ADME properties and the potencies of the interaction with the target in the MIE.

Several exposure metrics have been applied in toxicity studies of single chemicals.^35^ In the early days of aquatic toxicology, effects concentrations were estimated based on nominal concentrations administered to the test system. Test solutions were often renewed on a regular basis with the hope of maintaining constant exposure concentrations in studies with fish. Later on, ingenious dosing systems were developed for maintaining stable test concentrations.^36^ Nowadays, actual measured concentrations must be reported in aquatic toxicity studies according to the *ES&T* authors guidelines, but this information remains challenging and nonstandard for HT in vitro assays. Although there are some techniques available to measure chemical concentrations in small volume multiwell plate studies,^37,38^ they are not used on a routine basis. To further assist in predicting in vivo and in vitro exposures, there are several models to predict freely dissolved effect concentrations based on nominal doses,^39–41^ and cellular and membrane concentrations from freely dissolved concentration^13,42^ (Figure 3c).

Reverse dosimetry can also be applied in the quantitative extrapolation of in vitro-to-in vivo (qIVIVE) exposures.^43,47–49^ In qIVIVE, the effect concentrations in the medium or in cells from in vitro assays are applied as the in vivo plasma or tissue concentration in the AOP pathway (Figure 3d).^45^ A low percentile of the distribution of effect concentrations can then be converted by reverse PBK to safe oral equivalent dose (OED) or reference external dose (Figure 3d). Research supports extrapolating the unbound concentration in the medium of the in vitro test to unbound concentration in blood in the in vivo situation,^43,44,46^ because medium and blood binding is concentration-dependent, especially for anionic chemicals.^50^

The potential applications of qIVIVE models are numerous. For example, they can assist in the understanding of differences in species sensitivity by accounting for differences in ADME properties and sensitivity of the target among organisms. Importantly, qIVIVE is a tool that can link and harmonize human and environmental risks.

More in-depth studies that combine exposure and effects are the TK–TD models. A good example is the analysis of acute effects of the insecticide diazinon in two aquatic species.^24^ In this comprehensive study, overall effects were related to external and internal concentrations, the formation of the active metabolite and its reaction with the target. The TK–TD model could explain differences in species sensitivity. This highlights a mechanism-oriented study that combines properties related to ADME, internal exposure, MOA and interaction with a receptor in order to understand an AO. Such approaches represent an important path forward in toxicological studies.

### Importance of Understanding Mixtures

The AOP framework helps us to also better rationalize mixture effects. It is a central paradigm in mixture risk assessment that chemicals with the same mode of action act in a concentration-additive manner,^51^ and it has been proposed that mixture risk assessment should be applied along the entire AOP.^52^ This has been supported by experimental studies with complex mixtures that demonstrated that, especially at realistically low concentrations and effects, chemicals exert concentration-additive impacts on apical end points^53^ as well as in in vitro assays specific for MIEs and KEs.^33,54^

### Joining Forces: Integrating Ecotoxicology and Human Toxicology to Advance Research and Risk Assessment

An important nascent development in environmental and health sciences has been the “One Health” concept, which explicitly acknowledges the interactive, holistic nature of ecosystems from microbes to humans.^55^ In terms of risk assessment, One Health is predicated on consideration of chemical or nonchemical stressors in the context of their overall potential impacts from a systems perspective.^56^ One Health has significant ramifications in the field of toxicology and epidemiology that can utilize the concept in an immediate and pragmatic manner. Specifically, the biological pathways targeted by toxic chemicals often are highly conserved within definable taxonomic groups (e.g., endocrine systems in vertebrates) and, in some instances, across the entire biological spectrum in an ecosystem (e.g., basic energy metabolism). Consequently, pathway-based approaches to chemical safety assessment directly support the One Health concept. For example, an important component of AOP development is explicit definition of the taxonomic domain of applicability of a given pathway, which supports toxicological extrapolation across different species and test systems based on pathway conservation. Effects extrapolation across species is a prominent feature of the One Health paradigm, which has pragmatic implications in terms of increasing the efficiency of chemical safety assessments through use of data from NAMs. For example, due to significant conservation of key MIEs involving nuclear receptors (e.g., the estrogen receptor) data from mammalian-based HT assays can be used to effectively predict potential chemical effects in nonmammalian vertebrates.^57^ Exceptions can be pesticides that are designed to be taxa-specific.^58^

### Depth and Breadth—Not a Competition

The field has progressed significantly in the 20+ years since the first *ES&T* ecotoxicology special issue,^3^ especially in pathway-based toxicology. However, there remains the need within science for a deeper mechanistic understanding of chemical perturbation from the molecular to the ecosystem level. Various NAMs, including omics-based tools, are critical to achieving this broader understanding, although the hoped-for direct application of omics^5^ in risk assessment and regulation have not yet been fully realized. However, such information can at least be used in weight-of-evidence assessments supporting activities such as read across.^59^

A critical dilemma in the field is the need to better understand complex effects, including unexpected responses (e.g., on behavior) while being protective for the huge numbers of chemicals that have and will continue to emerge. The need to better understand complexity while simultaneously addressing the greater number of more than 340,000 chemicals in commerce^60^ will not be addressed through additional resources. This inherently requires acceleration, simplification and streamlining of approaches to define safe levels. Thus, we need to better prioritize existing resources such that chemicals of greatest potential concern are addressed.^61^

This is especially true in times where the progress in regulation has led to the phasing out of hazardous chemicals such as many chlorinated and brominated persistent organic pollutants (POP).^62,63^ The legacy PFAS (per- and polyfluoroalkyl substances) PFOS (perfluorooctanesulfonic acid) and PFOA (perfluorooctanoic acid) were listed for phaseout on the POP convention in 2009 and 2019, respectively,^64^ and have now been banned by most countries.^65^ As a consequence many replacement products have entered the global markets without proper safety assessments despite often having striking similarity to the chemicals they are replacing.^66^ The positive effects of voluntary restrictions and the subsequent phase-out of legacy PFAS sometimes have been offset by the increasing concentrations of alternative PFAS in marine wildlife and humans.^67^ Similarly, with the phasing out of the plasticizer bisphenol A (BPA), numerous replacement products have become available, many of which are not benign, causing the same type of endocrine disruption as BPA.^68,69^

### Outlook in Science and Regulation

Promising developments in the science of ecotoxicology have been addressed in this paper. Scientific progress has been made in several areas during the last decades. We foresee that the combination of research into AOPs and AEPs will provide opportunities to bring the fields of ecotoxicology and human health closer together into a One Health approach. The mutual exchange of experimental data in these two fields and the application of computational models will lead to a better understanding and foundation of environmental as well as human health-oriented quality criteria and standards.

There is also a strong link between AOPs and mixture assessments. We foresee that strengthening these connections will lead to a better understanding and prediction of effects of complex mixtures in the environment and to more appropriate new mixture indicators to quantify the exposome.

As we consider the future of regulatory toxicology, there is a substantial potential for artificial intelligence (AI),^70^ including novel machine and deep learning tools, to better organize and interpret existing data and fill data gaps by read across and unsupervised learning.^71^ For example, there currently is an effort through the European Commission’s Joint Research Centre to employ AI-based approaches for AOP development.^72^

Current AI approaches cannot achieve fully generative toxicity prediction because the available training data sets are neither sufficiently large nor consistently reliable. It has been estimated that experimental data covering approximately 1–10% of the chemical universe would be required to enable predictive modeling for roughly 8–46% of substances currently on the market.^73^ The chemical space—or at least our awareness of its breadth—has greatly expanded,^29^ but the research has tended to focus on a comparatively few high-visibility, easy-to-test chemicals.^74^ It is imperative that we expand the physicochemical space for hazard testing and improve screening methods to become more reliable.^13^ A recent analysis estimated that approximately 50% of ecotoxicological studies focused on only 65 environmental pollutants—an imbalance that is neither sustainable nor scientifically adequate for addressing the broader chemical landscape.^74^

The desire to work on “hot topics” has sometimes led researchers to lose sight of the ultimate goal of protecting human health and the environment from chemical effects. A reasonable agenda and strategy certainly entail in-depth detailed investigations for a limited number of reference chemicals but also must feature robust screening assessments of emerging (including replacement) chemicals. NAMs that are clearly anchored in defined MIEs and KEs will yield robust and reliable data for hazard assessment, support the development of sustainable chemicals by design (SSbD),^75^ and help find alternatives for essential uses.^76^ We know about the problem of PFAS: they are labeled “forever” chemicals due to high persistence, although their biological activity often is fairly nonspecific and may be predictable by baseline toxicity models. Many of them are surface active, capable of disturbing any membrane in any organism. In addition, many of them are anionic and capable of binding strongly to many types of proteins. If we accept that the physicochemical properties and reactivity/persistence of a chemical will play an important role for toxic potency, it will suffice to run a few basic NAMs covering essential AOPs for human health and environment to classify them as hazardous or not. We posit that it is unnecessary to evaluate every new PFAS in an unlimited number of sophisticated bioassays and animal tests to prove that they cause subtle chronic effects. For example, if we accept that immunotoxic effects of PFOA and PFOS are real,^77^ we do not need to wait for epidemiological evidence before we can categorize a new similarly structured PFAS or other chemical as potentially hazardous. Any chemical that is persistent and therefore has a long residence time in organisms and has the properties of being hydrophobic, anionic and/or surface active has a reasonable likelihood of producing different chronic effects. We have earlier called for a new approach to streamline and accelerate hazard assessment to assessment of “persistent toxicity” using NAMs.^78^ We should not delay the phase-out of persistent and toxic chemicals—including, but not limited to, PFAS—until chronic adverse effects in humans and wildlife become evident. Adverse effects observed in mammals—often detected early in marine mammals such as whales, which tend to accumulate particularly high body burdens of persistent toxic chemicals—are likely to emerge in humans as well, reflecting shared physiological pathways and susceptibility to bioaccumulative contaminants. Precautionary measures are justified when chemicals are identified as hazardous based on their intrinsic physicochemical characteristics and environmental persistence, particularly when this concern is reinforced by evidence from robust in vitro bioassays and mechanistically informed AOP analyses.

Levels of individual chemicals often have decreased, partially in response to phase-out of individual chemicals or improved wastewater technology, but at the same time the diversity and sheer number of chemicals produced and used has risen. Hence, the question today is not to protect the environment and human health from individual bad actors but from potentially harmful chemical cocktails. Real-world mixtures may be hazardous, even if individual mixture components would not elicit an effect on their own. NAMs can greatly accelerate the assessment of mixtures of chemicals in that they provide HT tools to evaluate complex and environmentally realistic mixtures.

Despite the scientific advances in the field of environmental toxicology, the lack of their translation into regulation and action has been the greatest impediment for reducing pollution impacts. Notably, the Intergovernmental Science-Policy Panel on Chemicals and Waste provides one opportunity to help address this challenge.^79^

## Supplementary Material



## Notes

Text S1: Analysis of publications in ES&T on Environmental Toxicology; Figure S1: Number of publications in ES&T from 1967 to 2025 (PDF)
