# Supplementary material for: The Role of ES&T in Advancing Environmental Toxicology and Chemical Risk Assessment: Past, Present, and Future
Source: Environ Sci Technol. 2026 Jun 1;60(23):16410–8. doi: 10.1021/acs.est.6c03315 (PMC13276907; doi:10.1021/acs.est.6c03315)
Supplement: Supplementary file 1 [file es6c03315_si_001.pdf]

Supporting Information

Perspective

The Role of ES&T in Advancing Environmental Toxicology and Chemical Risk Assessment: Past, Present, and Future

Beate. I. Escher[a,b,c]*, Joop L.M. Hermens[d], John P. Sumpter[e], Gerald T. Ankley[f]

[a]Department of Cell Toxicology, Helmholtz Centre for Environmental Research – UFZ, Permoser Str. 15, 04318 Leipzig, Germany

[b]Environmental Toxicology, Department of Geosciences, Eberhard Karls University Tübingen, Schnarrenberger Str. 94-96, 72076, Tübingen, Germany

[c]German Center for Child and Adolescent Health (DZKJ), partner site Leipzig/Dresden, Leipzig 04103, Germany

[d]Institute for Risk Assessment Sciences, Utrecht University, 3508 TD Utrecht, the Netherlands

[e]Brunel University London, Uxbridge, Middlesex, UB8 3PH, UK

[f]Integrated Biological Sciences, Department of Biology, University of Minnesota, Duluth,

MN, 55812, USA

*Corresponding author email address beate.escher@ufz.de

Two pages, one additional text, one Figure.

Table of content

Text S1: Analysis of publications in ES&T on Environmental Toxicology.

Figure S1: Number of publications in ES&T from 1967 to 2025.

Text S1: Analysis of publications in ES&T on Environmental Toxicology

We performed a bibliometric analysis using the Web of Science (https://www.webofscience.com), searching for (ES&T and (ecotox* or tox*)), (ES&T and ecotox*) or (ES&T and (tox* and human and health)).

Up until the mid-1990s, the toxicological impact of pollution was barely mentioned in published papers. Then, publications in this area started to rapidly increase, exceeding 200 toxicology-related or toxicology-motivated publications in 2010 (approx. 12% of publications in 2010) and rising up to 500 in 2025 (approx. 20% of total publications in 2025) (Figure S1). Overall, in the 60 years of ES&T 14% of all papers mentioned (ecotox* or tox*).

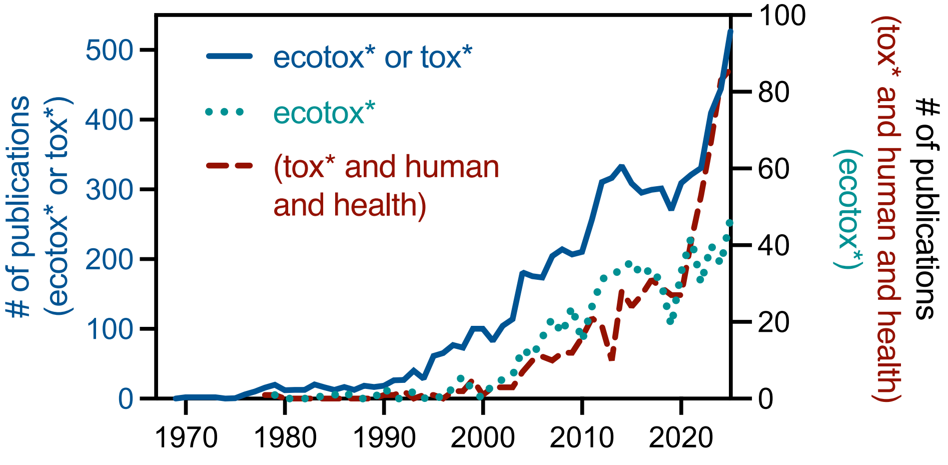

Figure S1. Number of publications in ES&T from 1967 to 2025 that had ecotoxicological or toxicological topics (ecotox or tox*) and the subset that only concerned ecotoxicology (ecotox*) or only human toxicology (tox* and human and health). Analysis using Web of Science (https://www.webofscience.com).*

Initially, toxicity considerations were only a motivation for environmental research but increasingly, they became central to it. After a fast increase in ecotoxicological topics, the publication numbers stagnated in this area in the years after 2010, although impact in form of citations continuously increased, while publication with topics (tox* and human and health) steadfastly increased in that time and overtook ecotoxicological topics in terms of number of publications and citations around 2020 (Figure S1).
